# Ultrasound-assisted Maillard reaction for the preparation of whey protein-fructooligosaccharide conjugates

**DOI:** 10.3389/fnut.2025.1531089

**Published:** 2025-05-09

**Authors:** Pallavi Singh, Anirban Roy, Aditi Kundu, Firoz Mondal, Mehulee Sarkar, Supradip Saha

**Affiliations:** ^1^Division of Agricultural Chemicals, ICAR-Indian Agricultural Research Institute, New Delhi, India; ^2^Division of Plant Pathology, ICAR-Indian Agricultural Research Institute, New Delhi, India

**Keywords:** whey protein concentrate, fructooligosaccharide, ultrasonication, Maillard reaction, response surface methodology

## Abstract

The current study investigated the effect of ultrasonication treatment on the conjugation of whey protein concentrate (WPC) with fructooligosaccharides (FOS) via the Maillard reaction (MR). The degree of glycation (DG) was evaluated by assessing the loss of amino groups using the o-phthaldialdehyde (OPA) method, and Maillard-type conjugation was confirmed through Fourier-transform infrared spectroscopy (FTIR) and sodium dodecyl sulfate-polyacrylamide gel electrophoresis (SDS-PAGE). Ultrasonication significantly accelerated the glycation reaction, achieving a glycation degree of 49.6% in just 60 min, compared to 12.4% over 16 h with conventional heating. This method produced Maillard-type conjugates with reduced browning intensity and improved, controlled protein solubility at acidic pH with higher oligosaccharide ratios, offering valuable potential for protein delivery applications. Furthermore, response surface methodology (RSM) was used for optimizing the Maillard reaction (MR) conditions for WPC and FOS.

## 1 Introduction

In the past few years, the growth of protein-saccharide conjugates has become a focus of research due to their potential to enhance protein functionality & broaden applications in the food industry. These conjugates enhance properties such as solubility, thermal stability, and emulsifying capacity, making proteins more versatile for various food applications (1). Among various modification techniques, protein conjugation with saccharides via the Maillard Reaction (MR) has gained significant attention. Unlike chemical modifications such as acetylation, deamidation, and succinylation, the MR is a naturally occurring process that involves the reaction between an amino group in amino acids or proteins and the carbonyl group of a reducing sugar or lipid peroxidation product (2). The MR progresses through three stages: initial, intermediate, and final. The initial stage of the reaction involves the condensation of the protein’s amino groups, primarily lysine, with the carbonyl group of a reducing sugar. This results in the formation of Schiff bases, which then rearrange to produce Amadori compounds. In the intermediate stage, these Amadori compounds degrade, leading to a diverse range of products. Finally, in the advanced stage, the reaction culminates in the formation of “melanoidins,” which are brown pigments responsible for the characteristic color and flavor of MRPs (3).

Numerous scientific investigations have proven that covalent attachment of proteins to saccharides, via the MR significantly enhances protein functionality without the need for additional chemical reagents (4, 5). This approach is widely seen as a safe and promising alternative in the food industry (6). The process is based on the Amadori rearrangement of MR, with factors like pH, time, temperature, and reactant ratios influencing the rate, extent, and attributes of the products and their functional properties (7). Studies have shown this modification improves protein emulsifying properties, solubility, and even antibacterial and antioxidant effects (8, 9). Compared to mono- and disaccharides, oligosaccharides conjugated with proteins yield more pronounced enhancements in physicochemical and functional properties (10). Glycation of proteins may also benefit individuals with food allergies by reducing IgE-binding capacity, making oligosaccharide-protein conjugation an effective strategy for modifying proteins in food applications (11).

Protein-polysaccharide conjugates are typically prepared using two traditional methods: dry heating and wet heating. The dry heating process consists of incubating the protein-polysaccharide mixture at a regulated temperature and humidity level over a prolonged period, which may vary from hours to several weeks. This method is time-consuming, often leading to excessive browning, and suffers from limited control over the reaction extent due to uneven contact between the reactants, making it less appealing from an industrial perspective (12). On the other hand, the wet heating process involves thermal treatment of the protein-saccharide mixture in a buffer solution for a few minutes to several hours (13). While this method significantly reduces reaction time, it still requires several hours to conjugate proteins with polysaccharides (14). Furthermore, at elevated temperatures or with extended processing times, protein denaturation and aggregation may occur, which can adversely affect the functional properties of the conjugates (15). Therefore, there is a need for innovative technologies to enhance the efficiency of the glycation between proteins and polysaccharides.

Several advanced techniques have been explored to accelerate the MR and overcome the limitations of traditional methods, including microwave (13), high pressure (16), radiation (17), dynamic high-pressure microfluidization (18), and pulsed electric fields (19). Ultrasound has also been utilized to enhance the glycation reaction; however, several studies have targeted on the interaction between amino acids and monosaccharides (20, 21), with fewer investigations on the conjugation between proteins and oligosaccharides. Li et al. 2013 (22) found that after wet-heating at 85°C for 22 h, the degree of glycosylation (DG) for protein-peanut isolate with dextran and protein peanut isolate with gum arabic conjugates were 35.6 and 31.5%, respectively, whereas ultrasound treatment for 40 min resulted in substantially higher DG values of 45.5 and 40.7%. Similarly, Mu et al. (23) and Li et al. (24) observed that ultrasound not only accelerated the glycation of soy protein isolate-Gum Arabic and peanut protein isolate- glucomannan but also improved the functional properties (like solubility and emulsifying capacity) of the conjugates compared to those produced by classical heating. Recently, Song et al. (25) reported, ultrasound-assisted treatment has been shown to improve the structural characteristics of *β*-lactoglobulin (*β*-LG) when complexed with hyaluronic acid (HA) at optimal pH and ratios, leading to areduction in random coils and *α*-helices and an increase in β-sheets. These changes enhanced the thermal stability, antioxidant properties, and functional performance of the complexes. Similarly, the ultrasound-assisted Maillard reaction has been employed by Song et al. (26) to develop *β*-LG-HA covalent complexes for curcumin (Cur) encapsulation, demonstrating superior antioxidant properties, enzyme inhibition activity, and controlled release under simulated digestion conditions. In addition, Li et al. (27) demonstrated that ultrasound-assisted complexation of *β*-lactoglobulin with neochlorogenic acid and cryptochlorogenic acid enhanced hydrophilicity, thermal stability, and bioaccessibility, offering potential for curcumin encapsulation.

The enhanced efficiency of the MR is attributed to the optimal mixing along with improved energy and mass transfer, achieved through ultrasound (21). Furthermore, it can induce changes in the secondary and tertiary structures of proteins, leading to a reduction in reaction time and an enhancement in the functional properties of the conjugates (23).

Fructooligosaccharides (FOS) are fructose-based oligosaccharides that are widely utilized as soluble dietary fiber because of their desirable sensory characteristics and low caloric content. These characteristics make them ideal for a range of food applications. Additionally, FOS are recognized as prebiotics, as they selectively promote the growth of beneficial gut bacteria, especially *Lactobacilli* and *Bifidobacteria*, highlighting their potential as key ingredients in functional foods aimed at improving digestive health (28). Whey protein concentrate (WPC), derived from cheese whey, is a valuable ingredient in the food industry owing to its impressive nutritional content and functional properties like emulsion stability and gel formation (29). It contains over 90% protein with minimal fat and lactose, making it an outstanding source of essential amino acids. However, its functional properties are sensitive to factors like pH, ionic strength, and temperature, which limit its versatility in various food systems and processing techniques (30). To improve the functional characteristics of WPC and broaden its applications, several methods, including glycation through the MR, have been explored (31, 32). While traditional dry and wet heating methods have been commonly utilized for this purpose, research on the effects of ultrasonication treatment on the glycation of WPC remains limited.

Due to advancements in statistical and mathematical techniques, response surface methodology (RSM) has become an effective tool for evaluating a broader range of experimental parameters, making it possible to optimize several factors and their interactions in relation to the response variables (33).

In this study, we used ultrasonication as a viable method for synthesizing MR products with a higher degree of glycation (DG) than classical heating methods. Our goal was to optimize the glycation process by using RSM to systematically assess the effects of critical processing parameters such as protein to oligosaccharide ratio, pH and reaction time on the synthesis of WPI-FOS conjugates. Further, the developed conjugate was characterized for its structural features via FT-IR and SDS-PAGE technologies. These findings may help enhance the applications of whey protein-based conjugates by tailoring glycation conditions for specific functional properties.

## 2 Materials and methods

### 2.1 Materials

WPC with 90% protein content was sourced from Davisco Foods International. Inc. (USA), o-Phthaldialdehyde (OPA) was purchased from the Hi-Media Laboratories Pvt. Ltd. FOS was extracted in laboratory from plantain peels according to method by Li et al. 2014 (34). Sodium tetraborate, *β*-mercaptoethanol, sodium dodecyl sulfate and all other chemicals involved were of analytical grade (Merck®India).

### 2.2 Preparation of the WPC-FOS conjugates

WPC (1% w/v) and FOS (2.5%w/v) solutions were prepared by dissolving each in deionized water with constant stirring for 2 h. The two solutions were then combined and stirred with a magnetic stirrer for an additional 3–4 h at room temperature. Following this, 100 mL of the resulting mixture was treated using ultrasonication equipment (VCX-750, 250 W, Sonics and Materials Inc., New town, USA) for 45 min at 70°C. The solution was rapidly cooled to room temperature in an ice bath after the reaction was completed and brown precipitates were formed which were then filtered and stored at 4°C for subsequent analysis. The degree of glycation was evaluated via RSM, investigating the effects of the WPC-FOS weight ratios (4:1, 2.5:1, and 1:1), pH values (9–11), and reaction times (30, 45, and 60 min). In addition, conjugates were also synthesized using conventional heating.

### 2.3 Determination of the degree of glycosylation (DG%)

The DG was determined by measuring the reduction in free amino groups using the o-phthaldialdehyde (OPA) assay (35). The OPA reagent was prepared by dissolving 80 mg of OPA in 2 mL of ethanol, then adding 25 mL of 0.10 M sodium tetraborate buffer (pH 9.5), 2.8 mL of a 20% (w/w) sodium dodecyl sulfate (SDS) solution, and 0.2 mL of *β*-mercaptoethanol. The mixture was then diluted to 50 mL with deionized water. A 0.2 mL sample solution (2 mg/mL protein) was mixed with 4 mL of the OPA reagent and incubated at 37°C for 5 min. Absorbance was measured at 340 nm using a UV–Vis double beam spectrophotometer (Motras Scientific Instruments Pvt. Ltd., Made in India). A blank was prepared using deionized water and the OPA reagent.

DG (%) was calculated as follow:


$$\begin{array}{l} DG=1-\\ \frac{\mathrm{concentration}\;\mathrm{of}\;\mathrm{free}\;\mathrm{amino}\;\mathrm{groups}\;\mathrm{after}\;\mathrm{conjugation}\;\left(M\right)}{\mathrm{concentration}\;\mathrm{of}\;\mathrm{free}\;\mathrm{amino}\;\mathrm{groups}\;\mathrm{before}\;\mathrm{conjugation}\;\left(M\right)}\times 100 \end{array}$$

### 2.4 Determination of browning index

The browning index of conjugates was measured following the procedure outlined by Qui et al. 2018 (36). To prepare the samples, they were suspended in 15 mL of phosphate buffer (0.2 M, pH 7). Then, 2 mL of this suspension was transferred into a tube, followed by the addition of 7 mL of 0.1% (w/v) SDS solution, and the mixture was thoroughly combined. The browning index was determined by measuring absorbance at 420 nm using a UV–Vis double beam spectrophotometer (Motras Scientific Instruments Pvt. Ltd., Made in India).

### 2.5 SDS-PAGE (sodium dodecyl sulfate polyacrylamide gel electrophoresis)

The analysis was conducted using a vertical gel electrophoresis unit (Bio-Rad, USA), following the method described by Liu et al. (37). The protein samples were diluted with sample buffer to 10 mg/mL and incubated in a water bath at 90°C for 5 min. A 5% stacking gel and 12% separating gel containing 0.1% SDS were prepared. Then, each sample mixture (15 μL) was loaded into the wells of the gel, and electrophoresis was performed at 80 V for 3 h in Tris-glycine running buffer (pH 8.2). Following this, the gels were stained with Coomassie Brilliant Blue R250 dye and destained using a mixture of 40% methanol and 10% glacial acetic acid.

### 2.6 FTIR spectral analysis

To examine the changes in the functional groups of WPC, FOS, and WPC-FOS conjugates, Fourier transform infrared spectroscopy (FTIR) spectra were recorded using an FTIR spectrometer (ALPHA, Bruker, Germany) operated in the ATR (attenuated total reflection) mode and coupled with OPUS processing software (version 7.0.122). The spectra were collected in the 600 to 4,000 cm^−1^ wave number range with a resolution of 4 cm^−1^, along with 256 scans.

### 2.7 Protein solubility analysis

For protein solubility analysis, 10 mg of WPC-FOS conjugates of different ratios (4:1, 2.5:1, and 1:1) were added to 1 mL of 0.1 M sodium acetate buffer and adjusted to pH 4, 7, and 9. The mixture was stirred at room temperature for 1 h and protein concentration was determined by the Bradford method (38) and calculated based on a standard curve of BSA.

### 2.8 Statistical analysis and experimental design

The Box–Behnken Design (BBD) was applied under the framework of response surface methodology (RSM) to optimize the conditions for the ultrasound-assisted Maillard reaction between WPC and FOS. Three independent variables—reaction time (30–60 min), pH (9–11), and protein-to-saccharide ratio (1:1–1:4)—were studied to determine their effects on the degree of glycation (DG). A total of 17 experimental runs were conducted, to assess the optimal conditions for the desired response. The independent factors and their levels are detailed in Table 1 and a second-order polynomial equation (Equation 1) was applied to fit the experimental data:


(1) $$Y=B_{0}+{\sum^{k}}_{i=1}\;B_{i}\;X_{i}+{\sum^{k}}_{i=1}\;B_{ii}{X^{2}}_{ii}+{\sum^{k}}_{i>j}\;B_{ij}X_{i}X_{j}+e$$

**Table 1:** Coded and actual variables utilized in the experimental design.

Coded variable levels			Independent variable
1	0	-1	
100	80	60	Time
9	6	3	pH
14	12	10	Mass ratio

Where Y denotes the response function (degree of glycation), B_0_ is the constant coefficient, and B_i_, B_ii_, and B_ij_ represent the coefficients for the linear, quadratic, and interaction terms, respectively. Obtained through polynomial regression. Here, e denotes the random error.

Analysis of variance (ANOVA) was used to determine the coefficients of regression, as well as interaction components. The experimental design matrix, data analysis, and optimization were conducted using Design Expert software (version 9.0.6.2) and the RSM-optimized results were validated through additional experimental data to confirm accuracy. Table 2 outlines the optimal factor design for three variables, along with the observed responses.

**Table 2:** Experimental design with three factors combinations.

S No.	Factor 1 X_1_: Time	Factor 2 X_2_: pH	Factor 3 X_3_: Mass ratio	Response Conjugation (%)
1	45	10	1:2.5	45.62
2	60	10	1:4	50.16
3	45	10	1:2.5	45.27
4	45	9	1:4	48.08
5	60	10	1:1	40.62
6	30	10	1:1	38.18
7	30	9	1:2.5	40.24
8	30	10	1:4	46.88
9	60	9	1:2.5	46.79
10	45	10	1:2.5	45.92
11	45	11	1:4	51.42
12	30	11	1:2.5	43.83
13	60	11	1:2.5	49.86
14	45	10	1:2.5	45.55
15	45	10	1:2.5	45.74
16	45	9	1:1	40.19
17	45	11	1:1	41.26

## 3 Results and discussion

### 3.1 Efficiency comparison and optimization of conjugate preparation: conventional heating vs. ultrasonication

As shown in Table 3, comparison between the efficiency of conventional heating and ultrasonication treatment for conjugate preparation by assessing the degree of glycosylation (DG%) over various time intervals was done. Conventional heating gradually increased DG from 5.77% at 4 h to 12.41% at 16 h, whereas ultrasonication achieved a significantly higher DG within a much shorter period, reaching 40.18% in just 30 min and 49.56% at 60 min.

**Table 3:** Comparison of DG Values for WPC-FOS conjugates produced by ultrasound treatment and classical heating.

Classical heating		Ultrasound treatment	
Time (h)	DG (%)	Time (min)	DG (%)
4	5.77	30	40.18
8	7.80	45	44.78
12	10.62	60	49.56
16	12.41		

This significant difference in efficiency can be explained by the mechanistic effects of ultrasonication compared to conventional heating. Conventional heating relies primarily on thermal energy to drive the reaction, often requiring prolonged exposure to high temperatures. This approach, while effective, can lead to several drawbacks, including excessive browning, protein denaturation, and loss of functional properties. Extended heating times also increase the likelihood of undesirable side reactions, such as the formation of advanced glycation end products. In contrast, ultrasound treatment utilizes cavitation effects, where high-intensity sound waves generate microscopic bubbles that collapse rapidly, creating localized regions of high temperature and pressure (23). This phenomenon enhances mass transfer and mixing efficiency, bringing reactants into closer proximity and facilitating faster reaction rates. Additionally, ultrasound disrupts protein structures, exposing reactive amino groups and making them more available for glycation (39).

As a result, ultrasonication-assisted glycation accelerates the MR, reducing the time needed to achieve high conjugation levels compared to conventional heating. So, based on these findings, we selected ultrasonication treatment for further optimization. Using RSM, we aimed to maximize DG% by adjusting three critical parameters: pH, reaction time, and the mass ratio of protein to oligosaccharide. This approach allowed for a systematic exploration of the effects and interactions of these variables, facilitating an efficient path to achieving the highest possible degree of glycosylation.

### 3.2 Model fitting and ultrasound assisted response surface optimization

Table 4 presents the coefficient of determination (R^2^), adj R-sq., lack of fit values, and *F*-values. In this model, terms such as A, B, C, AC, BC, A^2^, B^2^, and C^2^ were found to be significant. Terms with values above 0.1000 are considered insignificant. The high *F*-value of 208.52 indicates that the model is statistically significant (*p* < 0.001). The R^2^ value of 0.9963 shows that 99.63% of the variability is explained by the model, indicating a strong fit. The lack of fit F-value of 5.58 suggests no significant lack of fit when compared to the pure error. The 6.50% probability of such a large Lack of Fit F-value occurring due to random variation confirms the lack of fit is not significant, which is a positive outcome, implying the model fits the data well. The “Adeq Precision” ratio, which evaluates the signal-to-noise ratio, should be above 4 for optimal performance. In this study, the ratio of 60.33 confirms an adequate signal, showing that the experimental results are reliable and consistent. These tests underscore that the model accurately predicts the average results. So, the final fitted model is:


$$\begin{array}{l} \mathrm{Conjugation}=164.07319+0.568500\;A\\ –29.46458\;B+0.743611\;C+0.023333\;\mathrm{AB}\\ –0.035333\;\mathrm{AC}+0.728333\;BC–0.006039\;A^{2}\\ +1.39875\;B^{2}–0.580556\;C^{2}\text{.} \end{array}$$

**Table 4:** ANOVA analysis for the fitted model.

Source	Sum of Squares	df	Mean square	*F*-value	*p*-value	
Model	322.31	9	35.81	208.52	< 0.0001	*
A-Time	52.02	1	52.02	302.89	< 0.0001	*
B-pH	15.26	1	15.26	88.87	< 0.0001	*
C-Mass ratio	224.83	1	224.83	1309.06	< 0.0001	*
AB	0.4900	1	0.4900	2.85	0.1351	
AC	2.53	1	2.53	14.72	0.0064	
BC	4.77	1	4.77	27.80	0.0012	
A^2^	7.77	1	7.77	45.26	0.0003	
B^2^	8.24	1	8.24	47.97	0.0002	
C^2^	7.18	1	7.18	41.83	0.0003	
Residual	1.20	7	0.1717			
Lack of fit	0.9704	3	0.3235	5.58	0.0650	
Pure error	0.2318	4	0.0579			
Cor total	323.52	16				

**Figure 1:**
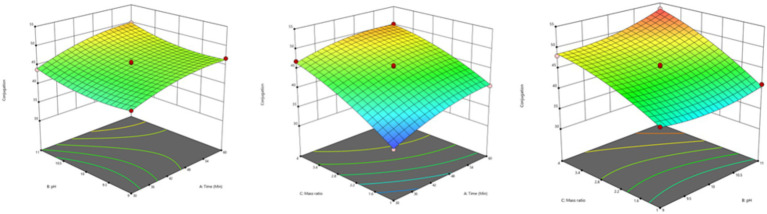
Response surface plots of independent variables for the degree of glycosylation (DG).

Impact of reaction parameters X_1_ (time), X_2_ (pH), and X_3_ (mass ratio) on response factor was thoroughly examined (Table 2). The response surface plots display the DG for protein-oligosaccharide conjugates formed by the MR under various conditions. The DG represents how extensively sugars have bonded to the protein molecules, which impacts the functional properties of the resulting conjugates, such as stability, solubility and emulsification. Figure 1 represents the mutual interaction between the DG and the reaction variable parameters (time, pH and saccharide to protein mass ratio). Increase in pH and prolonged reaction time enhance glycosylation by creating favorable conditions for the MR. A higher mass ratio of oligosaccharides provides more sugar molecules for binding, further increasing glycosylation. The effectiveness of the model in predicting DG was confirmed by these results. The profile for the optimal point revealed that the highest desirability level could be achieved with a protein-oligosaccharide ratio of 3.97:1, 47.5 min of heating at a pH of 10.97 (Supplementary Table S1). Under these optimized conditions, the predicted DG value was 53.62%, while the observed experimental value was 51.42%.

### 3.3 Effect of process parameters on DG

The DG (%) was assessed using the OPA assay, which quantifies the decrease in free amino groups per gram of protein during the MR. The DG values of the samples ranged from 38.2 to 51.4%. Time, pH, and the protein-oligosaccharide ratio were identified as significant factors in the model equation. The model accurately predicts the DG with 99.63% precision (Supplementary Table S2).

#### 3.3.1 Effect of initial pH

The DG rose rapidly as the pH increased from 9 to 11. This indicates that the initial pH of the system significantly impacts protein cross-linking through glycation, with the reaction proceeding more efficiently at elevated pH levels (40). This effect is due to pH influencing the proportion of amino acids in their unprotonated form, thereby enhancing the initial condensation phase of the MR at higher pH. Consequently, numerous studies on the MR between proteins/amino acids and oligosaccharides have been conducted under strongly alkaline conditions (41–43).

#### 3.3.2 Effect of WPC-FOS ratio

The WPC-FOS mass ratio varied between 1:1, 1:2.5, and 1:4. The highest conjugation percentage was observed at a 1:4 ratio (50.16%), and lowest at the 1:1 ratio (40.62%). The MR is primarily driven by the availability of free amino groups, which react with reducing sugars. Therefore, higher protein concentrations tend to favor the reaction, as they provide more amino groups for interaction with the saccharide. A similar finding was reported by Xue et al. (35) and Liu et al. (44) who observed that increasing the polysaccharide concentration accelerated the MR, leading to higher conjugation levels. Moreover, at high concentrations, FOS may hinder the MR due to steric effects and increase the viscosity of the reaction system, which can decrease the efficacy of ultrasonication treatment. This high viscosity limits the ability of ultrasonication to enhance mass transfer, disrupting the interaction between protein and saccharide and ultimately decreasing the glycation efficiency (21).

#### 3.3.3 Effect of time

The highest conjugation percentage was observed at 60 min of ultrasonication time (50.16%). The DG increased with sonication time from 30 to 60 min, as shown in Table 4. Similar findings were reported by Chen et al. (39) and Li et al. 2014 (24), who observed an enhancement in the glycation reaction with prolonged sonication. However, Corzo-Martínez et al. (21) reported a contrasting result, noting no significant increase in glycation and a marked rise in browning at higher sonication times. This discrepancy may be attributed to the elevated temperature during sonication, where cavitational bubbling could raise the local temperature, thereby reducing the overall efficiency of the reaction (45).

### 3.4 Browning intensity

**Figure 2:**
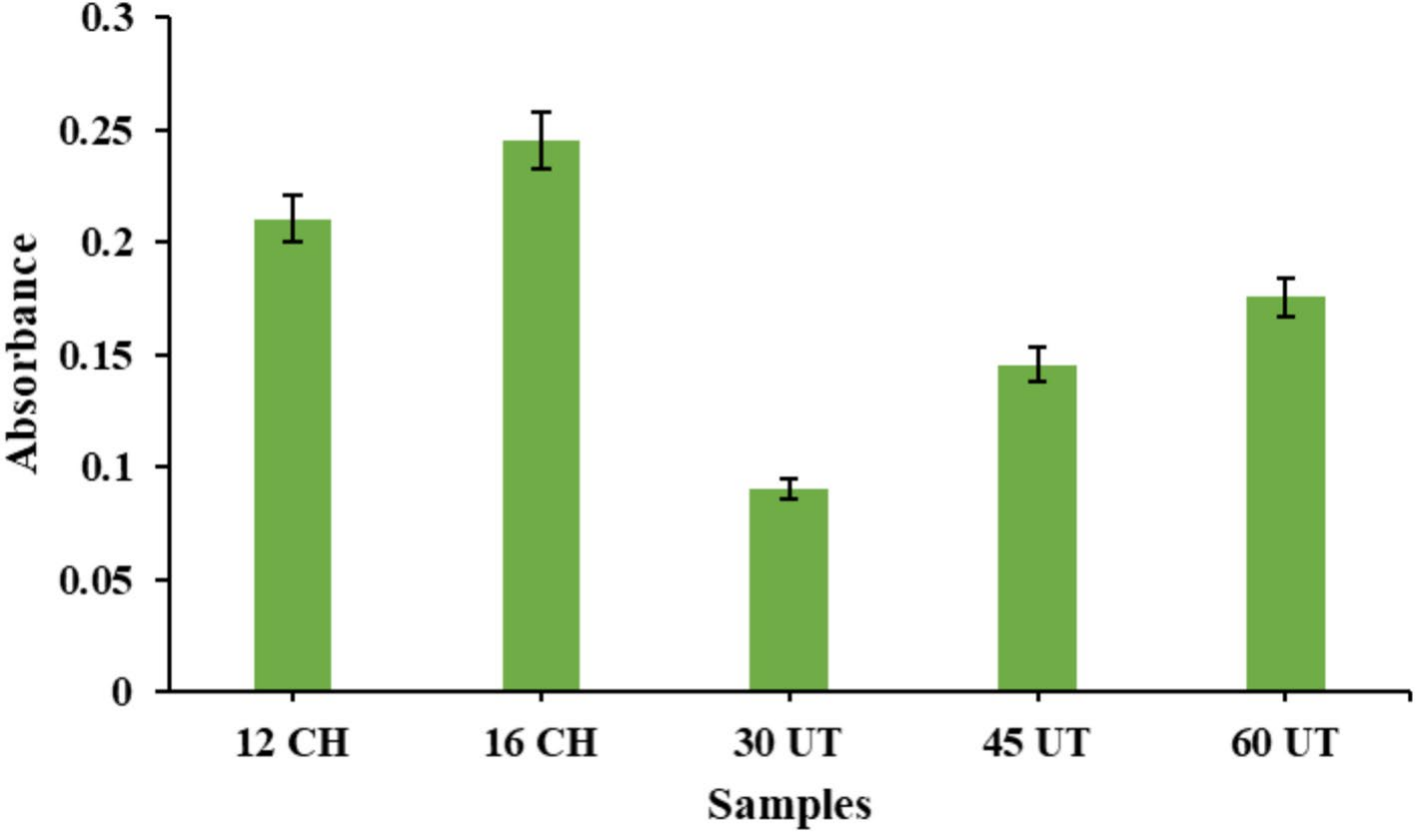
The browning intensity of WPC-FOS conjugates obtained at different time of ultrasonication and classical heating. 12 CH and 16 CH represents WPC-FOS conjugates obtained by classical heating for 12 h and 16 h whereas, 30 UT, 45 UT, and 60 UT 80 indicate the times (minutes) required to produce WPC-FOS conjugates by ultrasonication treatment.

The development of brown color appearance in the solution, caused by the formation of secondary MRPs, was evaluated. Browning is generally considered undesirable in food products and should be minimized. This color change is attributed to the breakdown of the Amadori product, a later stage in the MR, which can lead to the degradation of the protein-saccharide conjugate. Hence, controlling browning is crucial to maintaining the overall quality of the end product. As shown in the Figure 2 conjugates formed through conventional heating at 12 and 16 h exhibited a greater browning intensity compared to those prepared using ultrasound treatment at 30, 45, and 60 min. Our findings align with Zheng et al. (32), who indicated that ultrasound treatment minimizes side reactions during the grafting process. This occurs because ultrasound enhances the mixing and mass transfer, which reduces the formation of unwanted by-products and promotes more effective glycosylation between the protein and polysaccharide. This results in both faster MR and better control over browning and other undesired changes. Similarly, Zhao et al. (46) found that ultrasound treatment lowered the browning intensity of SPI/sugar Maillard reaction products (MRPs). They attributed this to ultrasound’s potential to hinder melanoidin formation by restraining the polymerization of intermediate substances. As a result, ultrasound not only accelerates the glycosylation of WPI and GA but also limits browning in the final products. However, contrasting results were seen in Wang et al. 2016 (47), where the browning intensity of BPI with glucose conjugates formed by ultrasonication was much higher than those prepared by traditional heating.

### 3.5 SDS-PAGE analysis

**Figure 3:**
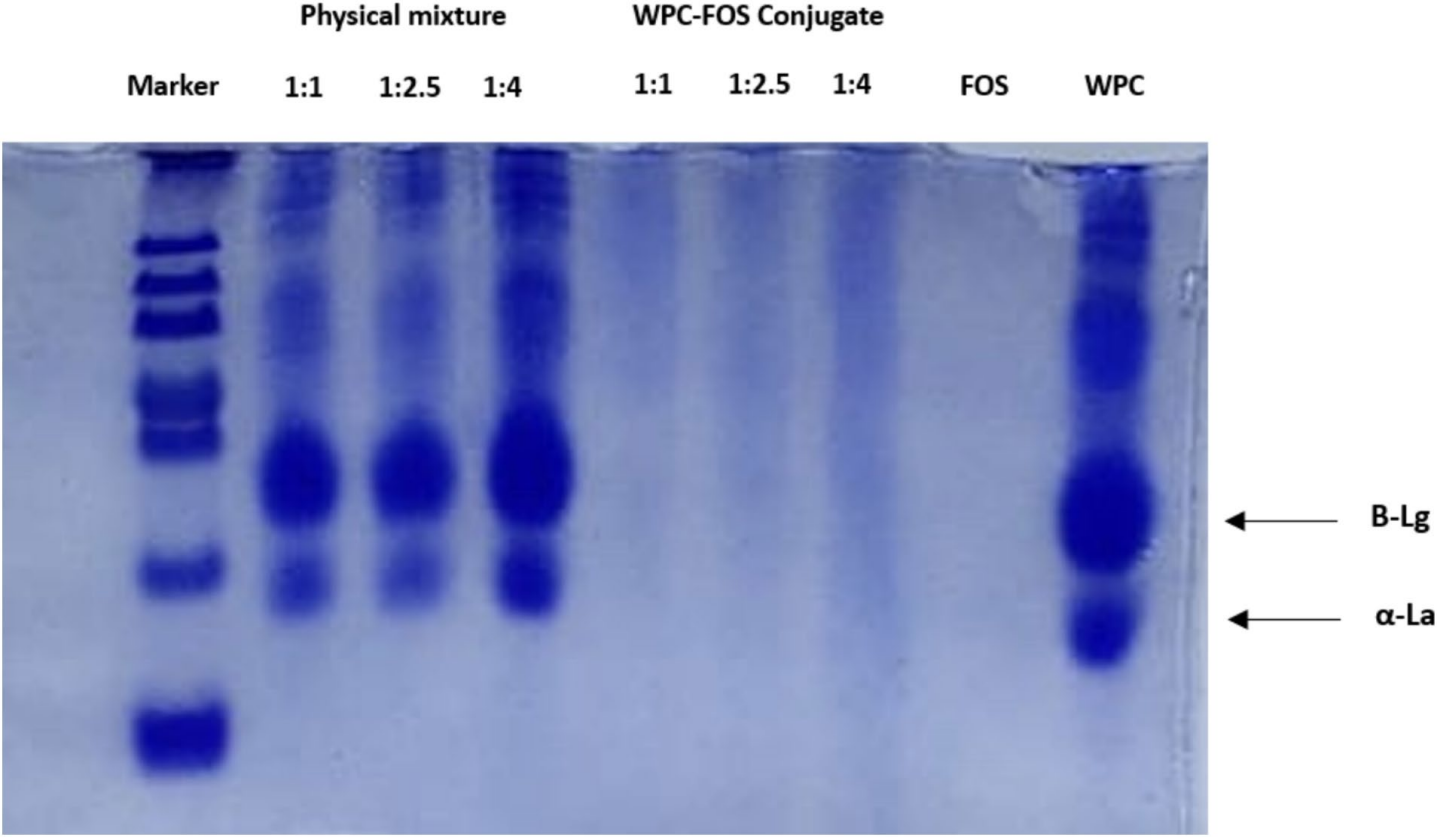
SDS-PAGE of WPC, FOS, physical mixture of WPC-FOS and WPC-FOS conjugates obtained at different mass ratios.

Figure 3 illustrates the electrophoretic patterns of WPC, FOS, and the conjugates subjected to different treatment conditions. In the SDS-PAGE analysis, the FOS lane shows no visible bands, as FOS alone lacks proteins, confirming it as a carbohydrate-only sample. In contrast, the WPC lane displays two distinct bands corresponding to *β*-lactoglobulin (*β*-Lg) and *α*-lactalbumin (*α*-La), which are characteristic of whey protein (48). In the physical mixture lanes (ratios 1:1, 1:2.5, and 1:4), the bands for *β*-Lg and α-La remain distinct, showing no interaction between WPC and FOS in the absence of covalent bonding. There was no significant band visible in the conjugate lane as because due to conjugation, size of the protein enhanced and it hinders in movement across SDS gel. No separate subunits are also present in the gel band. Very faint bands in the conjugate lane might be due to very limited unreacted proteins present in the mixture. Similarly, Ma et al. (49) reported that the ultrasound-assisted MR led to the emergence of new protein bands in SPI, along with the vanishing of some pre-existing bands. As WPC concentration increased, these larger protein-polysaccharide complexes became more prominent, supporting the formation of conjugates with reduced mobility due to their higher molecular weight. This pattern is consistent with the concept that higher molecular weight complexes, formed through conjugation, migrate more slowly in SDS-PAGE, confirming the effectiveness of the MR in generating WPC-FOS conjugates (50).

### 3.6 FT-IR analysis

**Figure 4:**
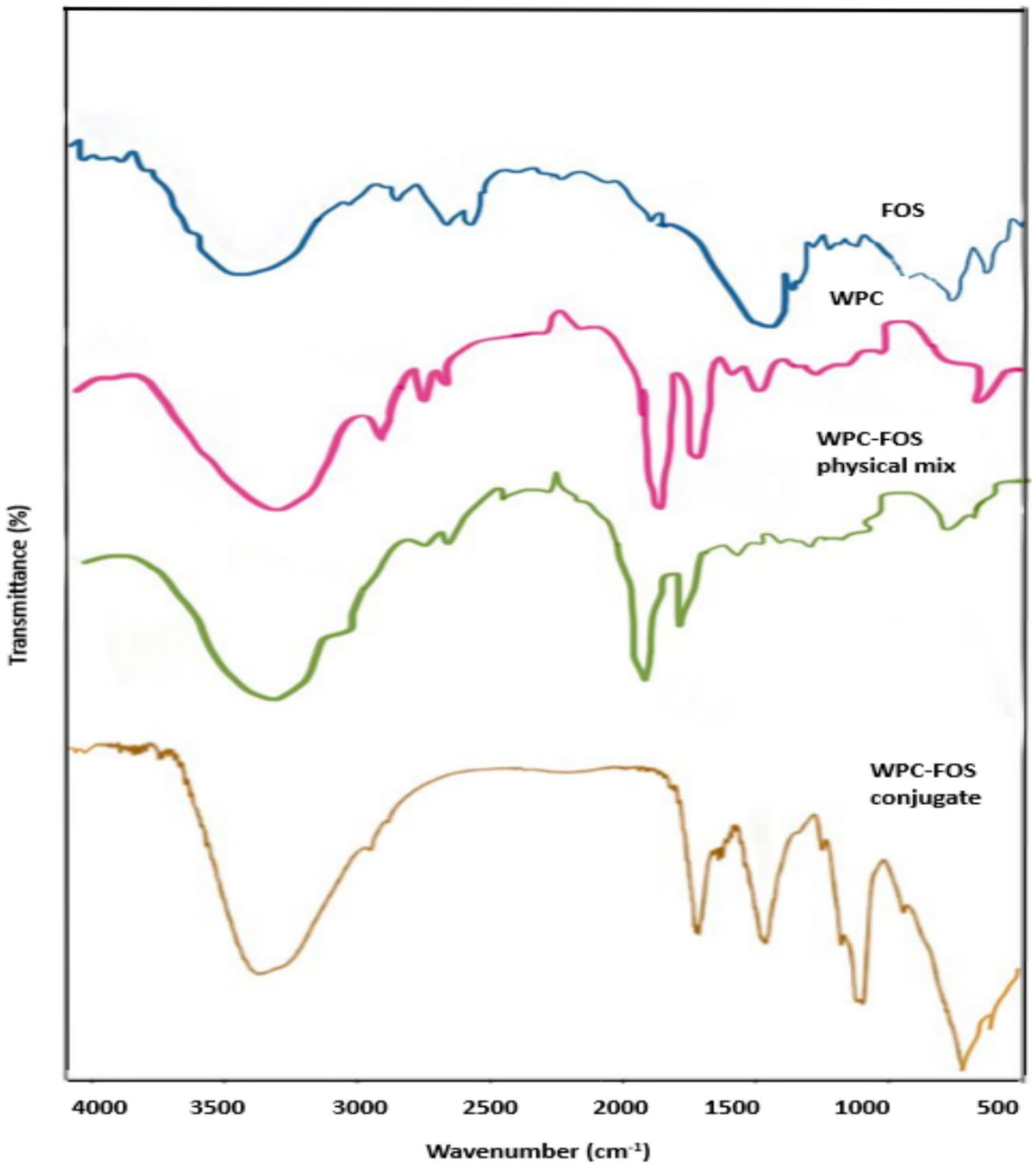
FT-IR spectra of WPC, FOS, WPC-FOS physical mixture and conjugates.

FTIR spectroscopy was employed to analyze the shifts and changes in absorption bands due to the conjugation between WPC and FOS, as shown in Figure 4. The characteristic protein bands for WPC appeared at 1643 cm^−1^ and 1,546 cm^−1^, representing the amide I and amide II regions, respectively, which are indicative of the protein structure (51). For FOS, peaks were observed at 3270 and 2,927 cm^−1^ corresponding to -OH and -CH functional groups, respectively. Additionally, the peak around 1,050 cm^−1^ was attributed to the vibration of aliphatic hydroxyl groups, while the peak around 870 cm^−1^ was characteristic of carbohydrate structures (52). In the physical mixture of WPC and FOS, the spectrum retained the distinct peaks of both WPC and FOS without significant shifts or changes in intensity. This indicates that no covalent bonds were formed between the components in the physical mix, and they maintained their original structures without new chemical interactions. In contrast, the FTIR spectrum of the WPC-FOS conjugate displayed noticeable changes. An increase in hydroxyl groups within the peptide chain was observed, along with the incorporation of more carbon–oxygen bonds, leading to enhanced absorption intensities in the corresponding regions. The -CH stretching vibrations of –CH_2_ and –CH_3_ groups in the saturated structure appeared between 3,100 and 2,850 cm^−1^ in the conjugate spectrum. Amadori products (C=O) and Schiff bases (C=N) are formed when WPC interacts with FOS. New peaks around 1,310 and 1,250 cm^−1^ appeared in the WPC-FOS conjugates probably due to covalent bonding between the free amino group of WPC and the carbonyl group at the FOS molecule’s terminal end. This interaction creates an -OH bending vibration within the saccharide ring (53). Furthermore, the absorptions of WPC-FOS conjugates in the 1,045–960 cm^−1^ region, due to side-chain vibrations of the protein (54), were stronger than those observed in WPI alone, indicating secondary structural modifications in the protein. These findings align with results from similar studies. Temenouga et al. (55), reported that covalent bonding between proteins and saccharides leads to a broad peak around 3,650–3,250 cm^−1^ and a new peak between 1,250–980 cm^−1^ due to the stretching vibrations of -OH and -CO bonds. Bonds. Additionally, Zhang et al. (56) observed significant structural modifications in protein-polysaccharide conjugates formed via Maillard reaction, with FTIR spectra showing new peaks around 3,100–3,480 cm^−1^ and 1,000–1,166 cm^−1^, indicative of enhanced hydrogen bonding and glycation-induced structural changes. Similarly, Khan et al. 2024 (57) reported that the Maillard reaction between pea protein and polydextrose led to conformational changes, including increased *β*-turns and random coils, which contributed to improved functional properties. These changes were validated through FTIR spectra displaying shifts in amide I and II regions and additional peaks associated with glycation product.

### 3.7 Protein solubility kinetics

Protein solubility, a key property influencing functional characteristics such as emulsifying and gelling, is crucial for protein applications. Calibration curve of bovine serum albumin (BSA) was prepared by using five different concentrations (50–300 μg mL^−1^) and the equation was derived as: *y* = 0.925x + 0.039.

**Figure 5:**
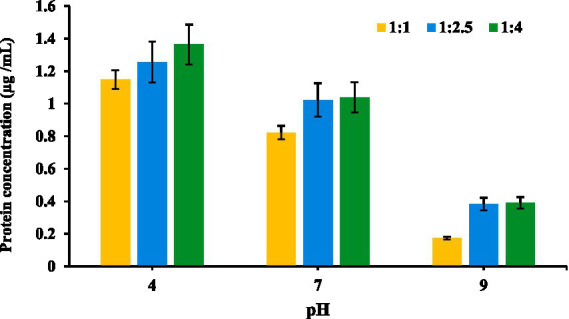
Solubilization of protein at different pH values.

Using the above equation the protein concentration at each pH was determined and the results as shown in Figure 5, which revealed that at acidic pH 4, protein solubility was highest, with all the three ratios. At neutral pH 7, protein solubility was moderate, reflecting stable but less disrupted protein interactions, while at alkaline pH 9, release was minimal, as proteins retained structure and exhibited strong binding within the conjugate.

Figure 5 shows that, protein solubility was highest for the 1:4 oligosaccharide-to-protein ratio and lowest for the 1:1 ratio. These findings align with previous research on MRs, which are widely explored for enhancing the functional properties of proteins, particularly solubility and surface stability (58). MR conjugates, formed by bonding proteins with saccharides, are known to improve protein stability and solubility under challenging conditions, such as low pH and high ionic strength, which can enhance emulsification properties (59).

Figure 5 illustrates how protein solubility varies in MR conjugates across different pH levels (4, 7, and 9) and oligosaccharide-to-protein ratios (1:1, 1:2.5, and 1:4). The data reveal that at acidic pH 4, protein solubility peaks, with the highest absorbance in the 1:4 ratio, followed by 1:2.5 and 1:1, which aligns with literature indicating that low pH promotes protein dissociation from conjugates due to weakened protein-carrier interactions and possible protein unfolding or hydrolysis (35). At neutral pH 7, release is moderate, reflecting stable but less disrupted protein interactions, while at alkaline pH 9, solubility is minimal, as proteins tend to retain structure and exhibit strong binding within the conjugate, limiting release. This pattern reflects that the acidic conditions enhance the stability and functionality of MRPs. Low pH environments promote covalent bonding between amino groups in proteins and reducing ends of oligosaccharides, which strengthens the conjugate structure and supports higher protein solubility (60). Moreover, higher oligosaccharide content, as in the 1:4 and 1:2.5 ratios, improves encapsulation and protein protection, enabling more controlled release. Thus, Maillard-type conjugates at acidic pH and higher oligosaccharide ratios are most effective for protein stability and release, proving valuable in controlled protein delivery applications.

## 4 Conclusion

The present study demonstrates the successful synthesis of WPC-FOS conjugates via Maillard reactions, with the conjugation optimized based on protein-to-oligosaccharide ratio, pH, and reaction time. The highest conjugation efficiency was achieved at a 1:4 mass ratio, pH 10.97, and a reaction time of 47.5 min, as determined through RSM. FT-IR and SDS-PAGE analysis validated the covalent bonding of FOS to WPC. Furthermore, ultrasonication treatment effectively accelerated the glycation rate between WPC and FOS, resulting in conjugates with significantly lower browning intensity and higher degrees of glycation compared to those prepared by conventional heating. These improvements are attributed to structural modifications induced by ultrasound, which enhance mixing efficiency and facilitate glycation. However, this study has certain limitations. While the degree of glycation and structural modifications were evaluated, a detailed analysis of the reaction kinetics and underlying molecular interactions was not performed. Additionally, the stability and functionality of the conjugates under various processing and storage conditions, critical for their practical application in food systems, were not explored. Lastly, the scalability of the ultrasound- assisted process was not investigated, which is essential for industrial applications.

Overall, our findings suggest that protein-oligosaccharide conjugates formed through Maillard reactions, particularly via ultrasound-assisted treatment, offer a promising method to enhance functional properties and broaden applications in the food industry. These conjugates could serve as effective encapsulation substances that protect bioactive compounds from external environmental factors. Future research should address the limitations outlined above to further elucidate the glycation mechanism, optimize the ultrasonication process, and validate the industrial scalability and stability of the conjugates.

## Data Availability

The original contributions presented in the study are included in the article/Supplementary material, further inquiries can be directed to the corresponding author.
