# Supplementary material for: Ultrasound-assisted Maillard reaction for the preparation of whey protein-fructooligosaccharide conjugates
Source: Front Nutr. 2025 May 9;12:1531089. doi: 10.3389/fnut.2025.1531089 (PMC12101087; doi:10.3389/fnut.2025.1531089)

SUPPLEMANTARY INFO


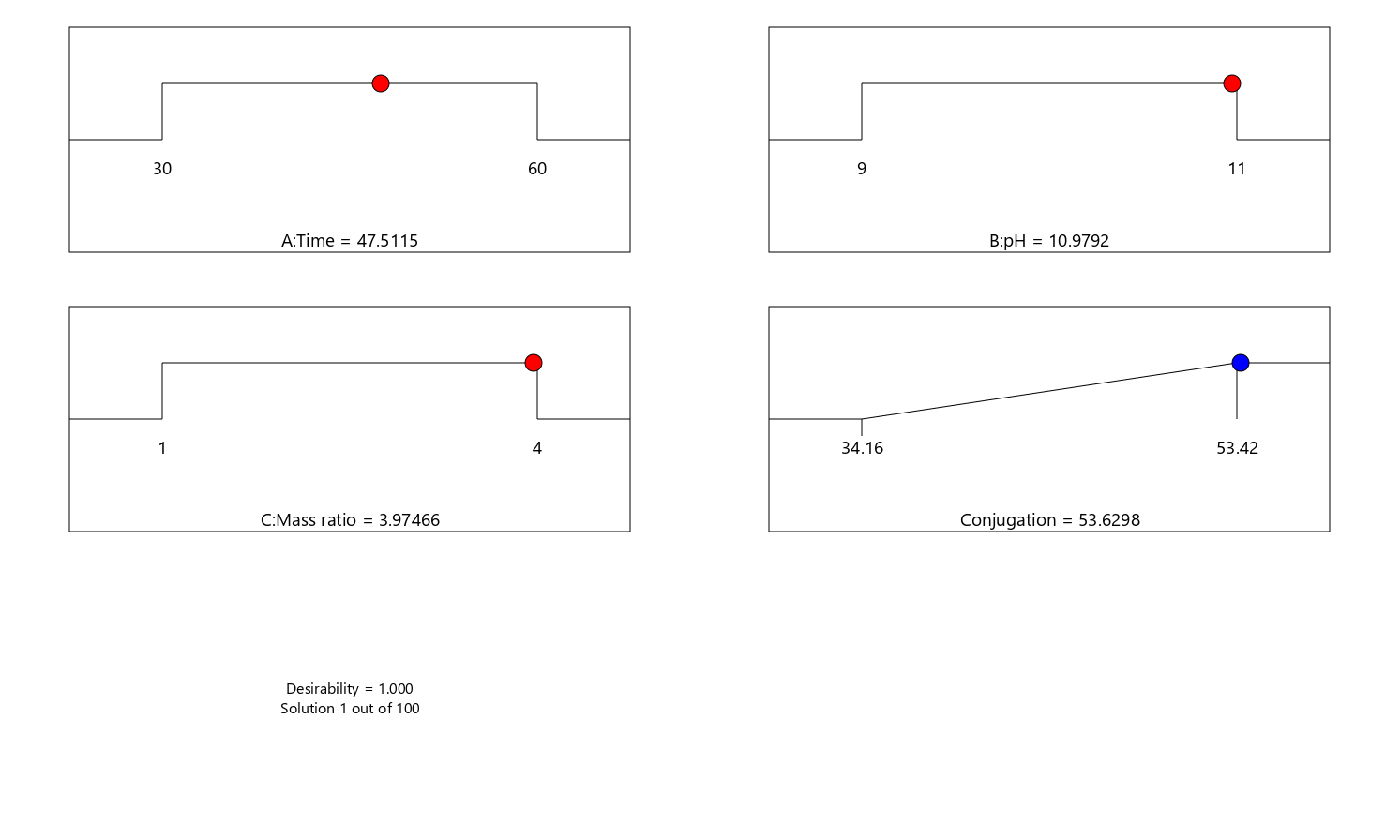


**Table S1**

| **Std. Dev.** | 0.4144 |  | **R²** | 0.9963 |
| --- | --- | --- | --- | --- |
| **Mean** | 45.02 |  | **Adjusted R²** | 0.9915 |
| **C.V. %** | 0.9204 |  | **Predicted R²** | 0.9509 |
|  |  |  | **Adeq Precision** | 60.3394 |

**Table S2**

**Supplementary info**

Full scanned image of gel


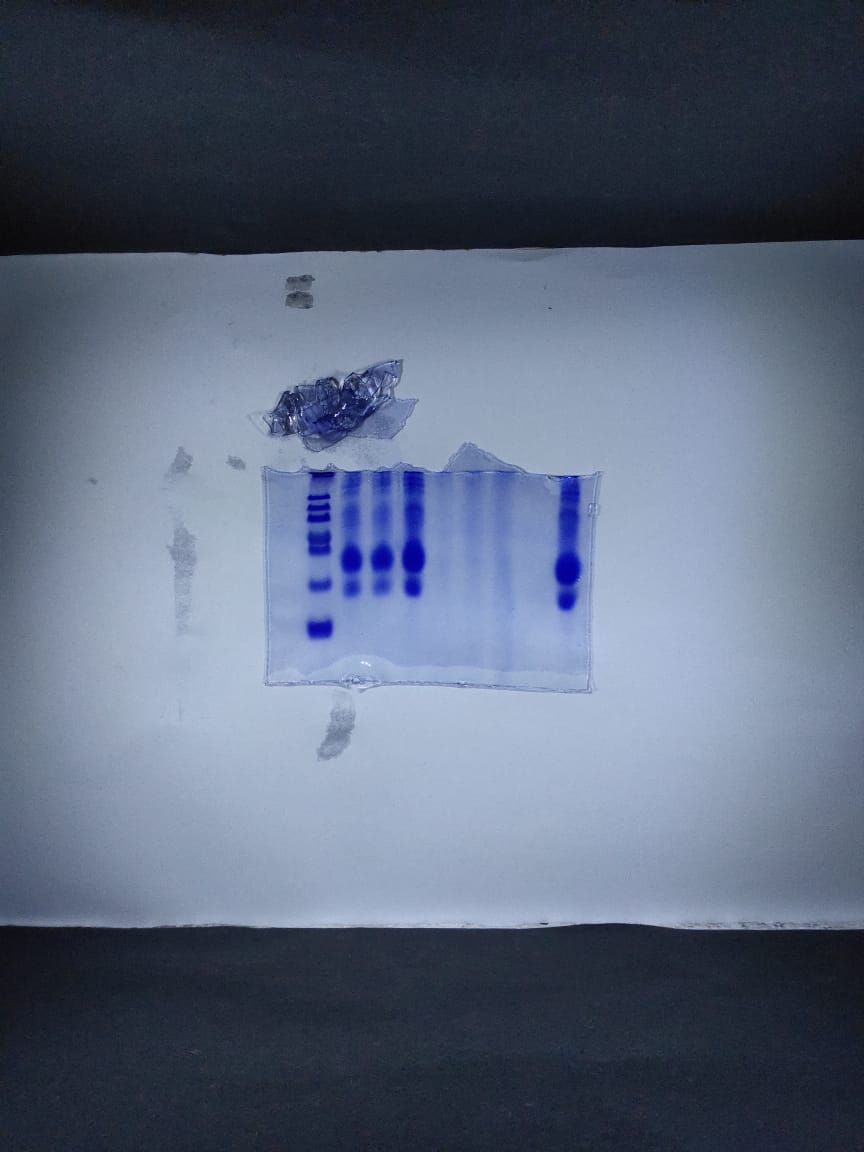


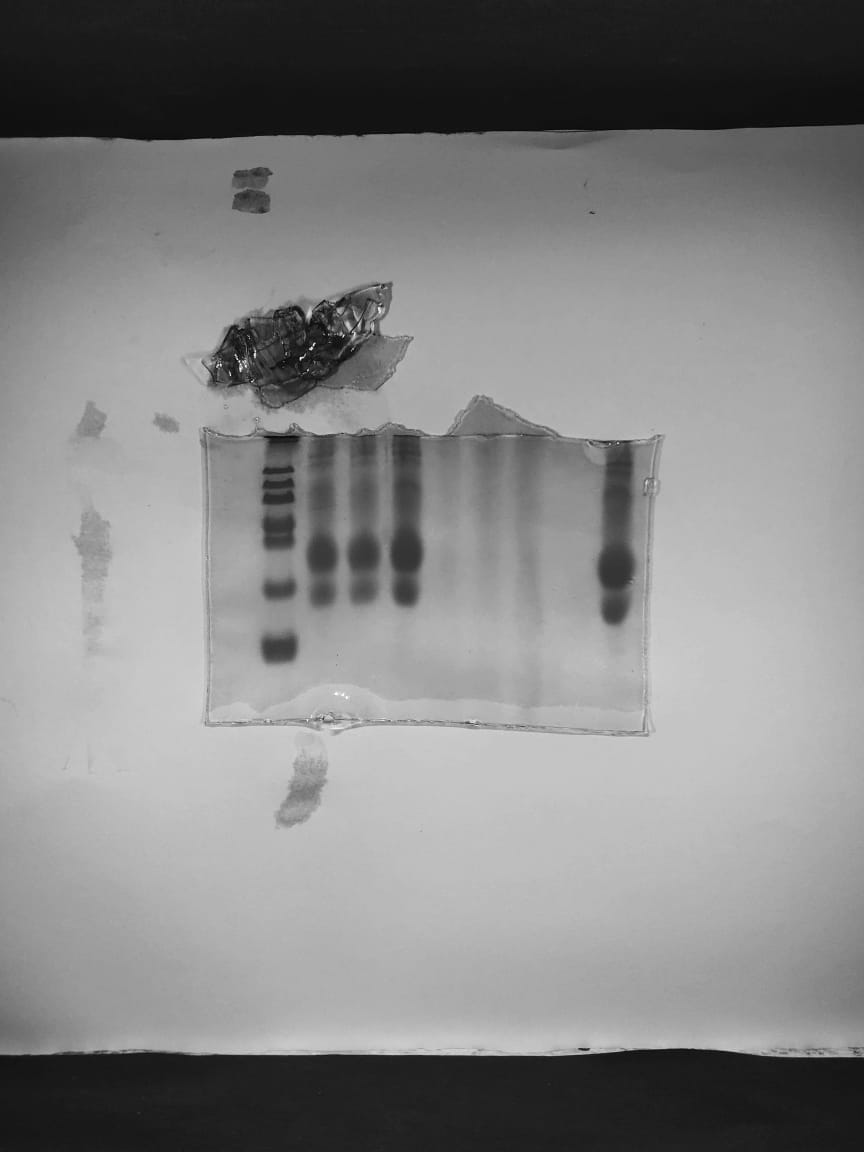

Supplement: Supplementary file 1 [file Table_1.docx]
